# Escalation to transplantation in refractory stone heart syndrome: a case report of advanced mechanical circulatory support failure

**DOI:** 10.1186/s13019-026-04455-5

**Published:** 2026-06-16

**Authors:** Theresa Holst, Jasmin Hanke, Reza Poyanmehr, Martin Hinteregger, Jan D. Schmitto, Günes Dogan, Murat Avsar, Alexander Weymann, Arjang Ruhparwar, Bastian Schmack

**Affiliations:** Department of Cardiac, Thoracic, Transplantation and Vascular Surgery, Hannover Medical School, Carl-Neuberg-Straße 1, 30625 Hannover, Germany

**Keywords:** Open heart surgery, Cardiopulmonary bypass, Stone heart syndrome, Temporal mechanical circulatory support, Heart transplantation

## Abstract

**Background:**

Stone heart syndrome was first described by Denton Cooley in 1972 and represents an extremely rare but severe complication following cardiac surgery. The condition is characterized by an irreversible, concentric, spastic contracture of the myocardium occurring during reperfusion which does not respond to either pharmacological or mechanical interventions. The underlying pathophysiology remains poorly understood, but an association with suboptimal cardioprotection and prolonged ischemia time has been suggested.

**Case presentation:**

A 22-year-old otherwise healthy patient was admitted for elective mitral valve surgery due to severe mitral regurgitation caused by bileaflet prolapse and a history of cardiomyopathy of unclear etiology. Upon weaning from cardiopulmonary bypass, the patient developed biventricular heart failure requiring veno-arterial extracorporeal membrane oxygenation. Transesophageal echocardiography showed a stone heart. In the following days, temporary mechanical circulatory support was escalated to veno-arterio-venous extracorporeal membrane oxygenation and Impella. On postoperative day 5, a durable left ventricular assist device was implanted. Due to refractory stone heart syndrome, the patient was evaluated for heart transplantation which was successfully performed on postoperative day 23. The subsequent course was largely uneventful. The patient was discharged to rehabilitation at 6 weeks post transplantation.

**Conclusion:**

Acute terminal heart failure due to perioperative stone heart is rare. In cases of irreversible global myocardial injury, orthotopic heart transplantation remains the only curative treatment option.

## Background

Stone heart syndrome was first described by Denton Cooley in 1972 [1]. It represents an extremely rare but severe complication following heart surgery involving cardiopulmonary bypass in both children and adults and accounts for approximately 2% of perioperative deaths [2]. It is characterized by an irreversible, concentric, spastic contracture of the myocardium occurring during reperfusion after cardioplegic cardiac arrest and leading to acute myocardial insufficiency that does not respond to either pharmacological or mechanical interventions. Myocardial hypertrophy with diffuse fibrosis is considered a predisposing factor, although the underlying pathophysiology still remains poorly understood [1, 3, 4]. Stone heart syndrome is considered a dreaded complication as outcomes remain fatal in most cases [2]. We herewith report the case of a patient who was successfully managed by temporary mechanical circulatory support followed by heart transplantation. To our knowledge, only one similar case has been described in literature in which an intracorporeal abdominal left ventricular assist device (LVAD) was used as a bridge-to-transplant. That individual, however, did not survive the early postoperative period despite successful transplantation [5, 6]. 

## Case presentation

A 22-year-old male, otherwise healthy patient with a low perioperative risk (EuroSCORE II 0.75%) was admitted to our center in February 2025 for elective surgery for severe mitral regurgitation caused by bileaflet prolapse. The patient had been closely monitored by cardiologists specialized in adult congenital heart disease since 2020, when he was first hospitalized for evaluation of markedly enlarged left ventricular (LV) diameters without significant valvular dysfunction. At that time, a myocardial biopsy demonstrated moderate myocardial damage, perivascular fibrosis and mild lymphocytic myocarditis leading to the diagnosis of cardiomyopathy of unknown etiology. Other cardiac and non-cardiac comorbidities were ruled out. In the following years, LV dimensions remained largely stable and ejection fraction preserved, although the degree of mitral regurgitation progressively worsened. Simultaneously, dyspnea increasingly aggravated. Therefore, indication for corrective surgery was established by interdisciplinary consensus. Preoperative repeat myocardial biopsy to reassess the underlying myocardial substrate was not performed.

Minimally-invasive mitral valve (MV) surgery was performed via lateral mini-thoracotomy. Myocardial protection was achieved using singe-dose antegrade administration of Bretschneider histidine-tryptophan-ketoglutarate (HTK, Custodiol^®^) cardioplegia after aortic cross-clamping. In the absence of aortic regurgitation and coronary obstruction, cardioplegia was delivered via aortic root cannula to ensure homogeneous coronary distribution. Systemic mild hypothermia (32 °C) was maintained throughout cardiopulmonary bypass to reduce myocardial oxygen consumption. Meticulous attention was paid to avoid LV distention through appropriate LV venting. To avert cardioplegia washout, periprocedural water testing of MV competency was performed using another dose of HTK cardioplegia instead of classical saline.

**Fig. 1:**
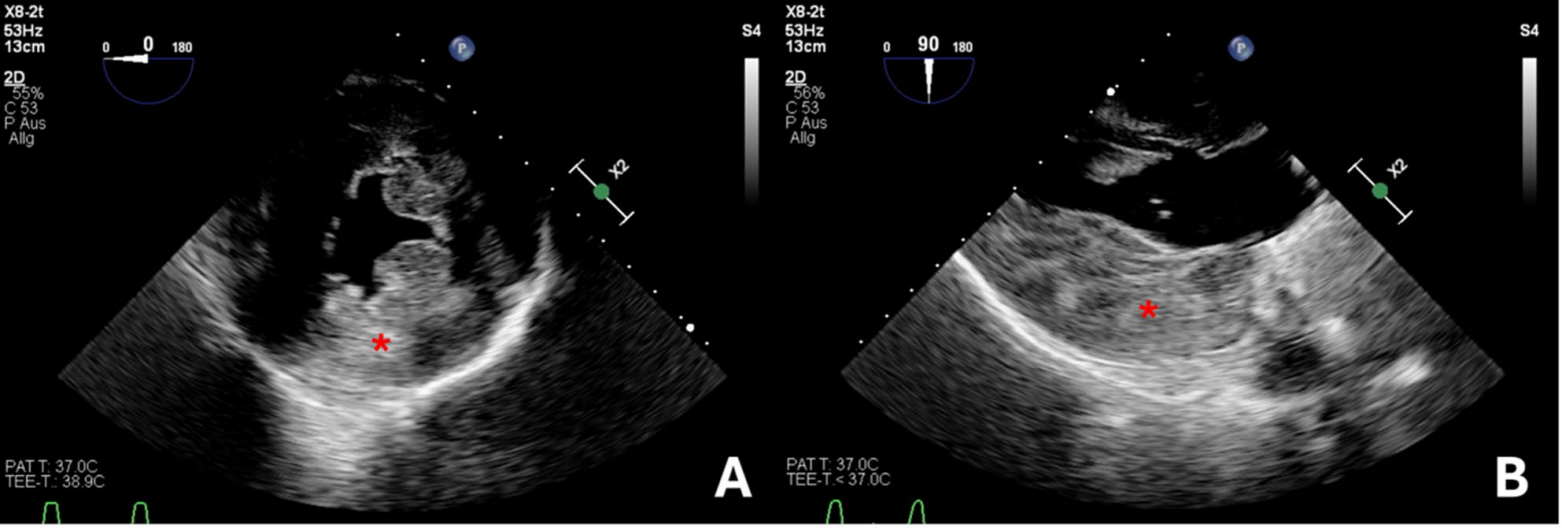
Stone heart in transgastric mid-papillary short-axis (**a**) and long-axis (**b**) views. Red asterisk: markedly thickened (i.e., contracted) left ventricular myocardium

**Fig. 2:**
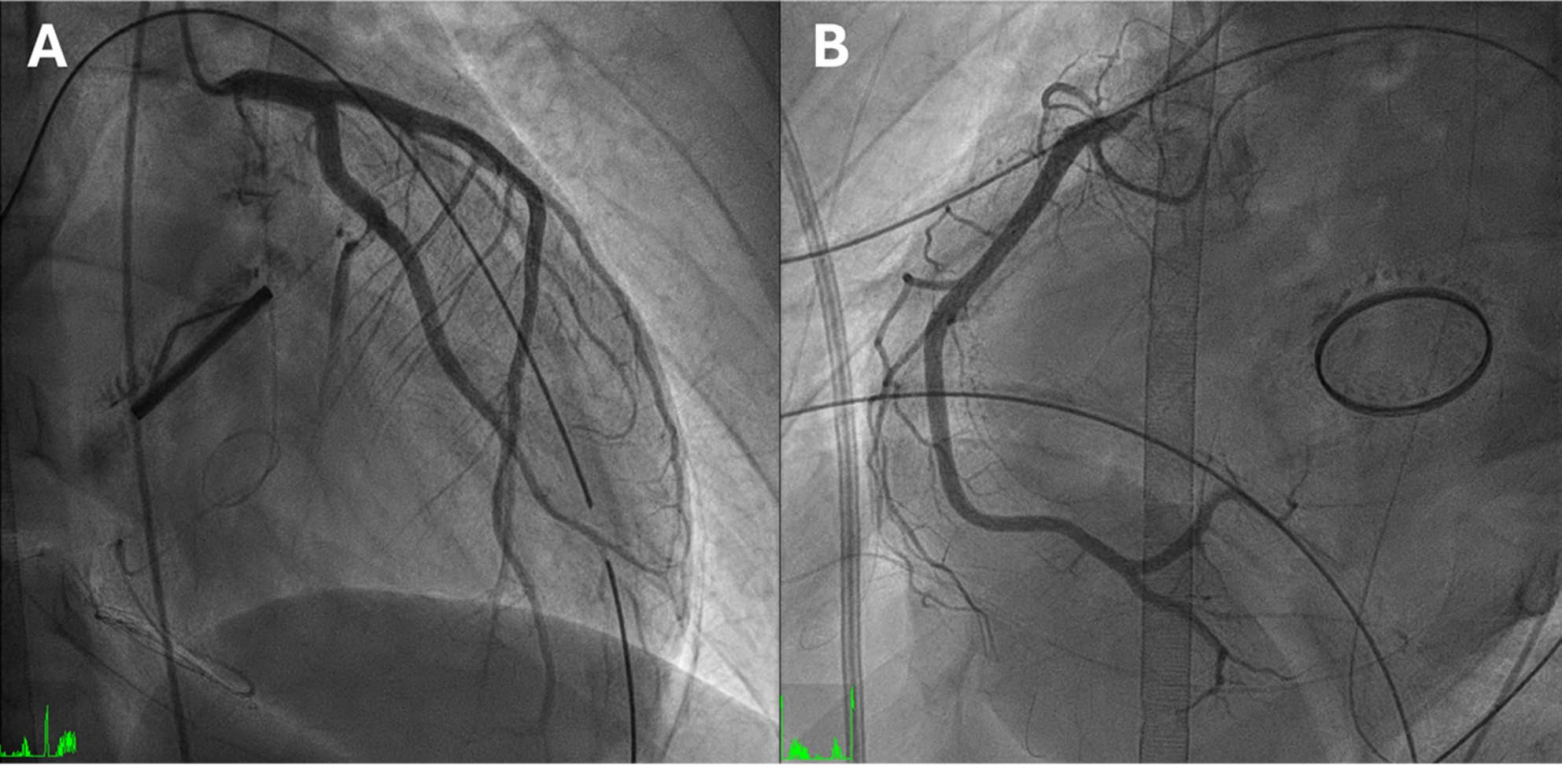
Angiography excluding a reversible perfusion deficit of the left (**a**) and right (**b**) coronary artery

**Fig. 3:**
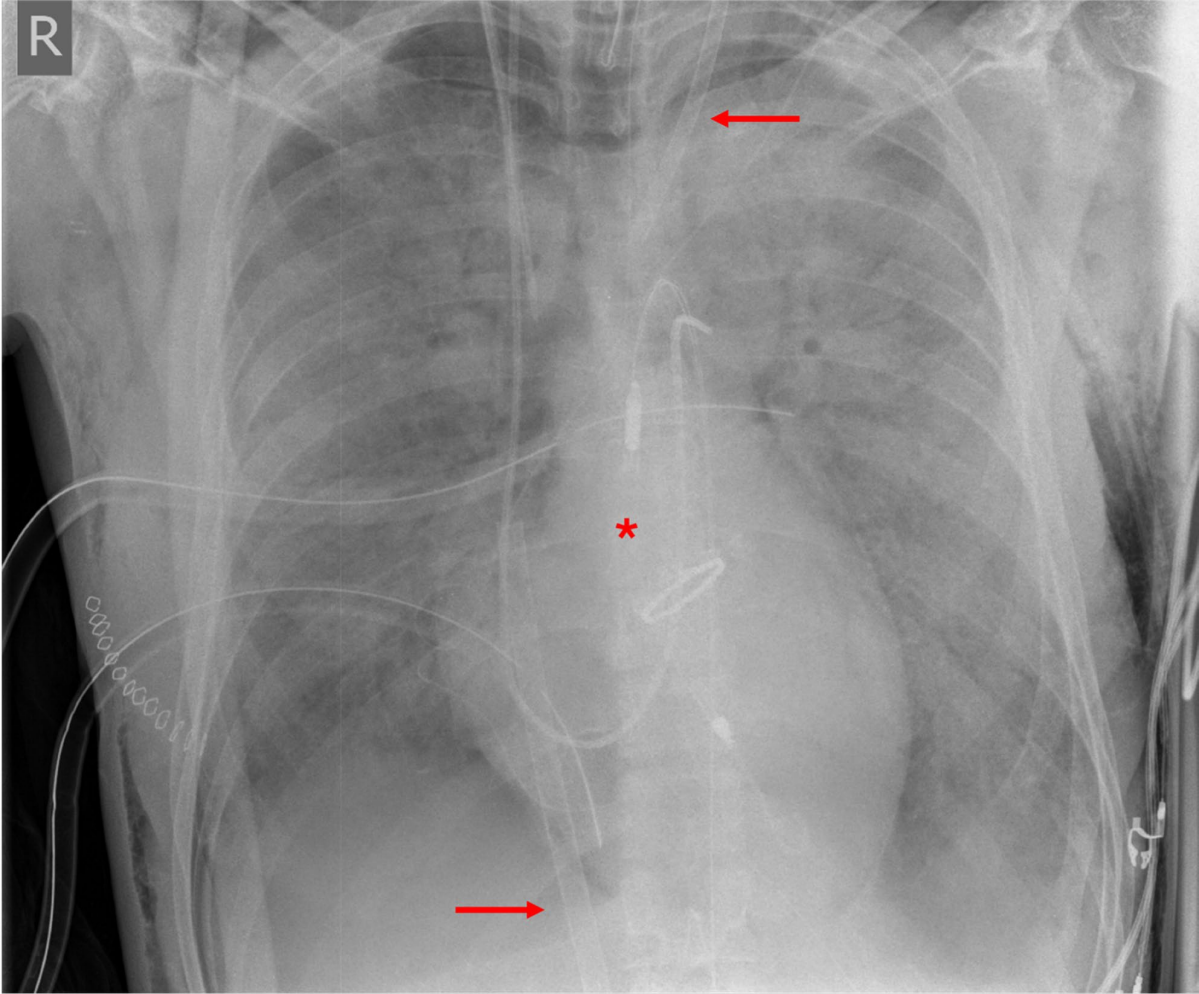
Progressive pulmonary edema despite veno-arterio-venous extracorporal membrane oxygenation and Impella CP (vav-ECMELLA) support. Red arrows: femoral venous drainage and additional jugular venous return cannula; red asterisk: Impella pump

Intraoperative inspection of the MV revealed severe morphological degeneration of the MV with myxomatous thickening of both leaflets and pronounced annular calcifications involving the P1/2 and A2/3 segments. After an initial attempt to repair the altered valve, a suboptimal result was seen prior to reperfusion. In the absence of further repair options, conversion to MV replacement was decided on and a 33 mm mechanical heart valve prothesis (St. Jude Medical Masters HP, St. Paul, Minnesota, USA) was implanted during the same cardioplegic arrest. Total aortic cross-clamp was 166 min. Upon weaning from cardiopulmonary bypass, the patient presented severe biventricular failure. Transesophageal echocardiography demonstrated a stone heart (see Fig. 1) which persisted even despite prolonged reperfusion efforts, necessitating the switch to veno-arterial extracorporeal membrane oxygenation (va-ECMO) via femoral access. A reversible coronary perfusion deficit as underlying cause was excluded by intraoperative coronary angiography (see Fig. 2). After va-ECMO initiation and chest closure, the patient was transferred to intensive care demanding high-dose inotropic and vasopressor support. The following day, an Impella CP (Abiomed, Danvers, MA, USA) was implanted via femoral access to allow for adequate LV unloading in the presence of a mechanical mitral valve and resulting LV low-flow condition (va-ECMELLA). We intentionally opted for the Impella CP because of the small LV cavity and the risk of suboptimal device positioning and interference with intracavitary structures associated with other Impella types. Due to rapidly progressive respiratory failure caused by severe pulmonary edema, an additional jugular venous return ECMO cannula was inserted (vav-ECMELLA) on postoperative day (POD) 3 to ensure adequate oxygenation and reduce pulmonary stress. As multiple vav-ECMELLA weaning attempts failed and pulmonary edema progressively worsened (see Fig. 3), a permanent (LVAD; HeartMate 3, Abbott, Pleasonton, CA, USA) as bridge-to-recovery was implanted on POD 5. Comprehensive histopathological and molecular biological assessment of the tissue obtained from the resected native myocardium at the site of LVAD inflow cannula insertion demonstrated mild myocardial inflammation and fibrosis (i.e., inflammatory cardiomyopathy), but no evidence of active myocarditis according to the Dallas classification, clinically relevant cardiotropic viral infection, cardiac amyloidosis or any other storage disease. Vav-ECMELLA support was continued until POD 8, when respiratory function had sufficiently recovered to remove the additional jugular venous return canula. Va-ECMO support could be discontinued on POD 10. However, LV function remained severely impaired on LVAD support with no evidence of myocardial recovery on serial assessments and continued dependency of high-dose inotropic medication. Given progressive end-organ dysfunction as well as the patient’s young age and absence of significant preoperative comorbidities, a comprehensive evaluation for high-urgency heart transplantation was initiated and the patient was finally deemed suitable and listed on day 15 after MV surgery. A suitable donor organ was allocated on POD 23 and orthotopic heart transplantation was successfully performed using the bicaval technique. Post-transplant histopathological assessment of the explanted native heart did not reveal any additional abnormalities and was fully consistent with the results obtained from the earlier myocardial tissue analysis.

The further postoperative course was largely uneventful apart from right-sided chronic pleural empyema requiring video-assisted thoracoscopy, left-sided deep vein thrombosis requiring oral anticoagulation as well as critical illness polyneuropathia with unilateral peroneal nerve palsy requiring intensive physical therapy. On day 65 after MV surgery, the patient was discharged to rehabilitation in a good general condition.

At last follow-up visit in November 2025, myocardial biopsy ruled out acute cellular rejection. Moreover, transthoracic echocardiography confirmed satisfactory allograft performance with preserved systolic and diastolic biventricular function and normal wall motion. Consistent with these findings, the patient reported excellent overall health, close to normal physical capacity, no increased susceptibility to infections, normal appetite and a stable body weight.

## Discussion and conclusion

Acute terminal heart failure due to perioperative stone heart syndrome is rare, but almost uniformly fatal because of irreversible global myocardial injury of unknown causal origin [2]. 

An association with suboptimal cardioprotection and prolonged ischemia time during cardioplegic cardiac arrest has been suggested and patients with preexisting cardiomyopathy may be particularly prone. Hence, prevention relies on effective and homogeneous cardioplegia delivery with re-dosing at appropriate intervals during prolonged procedures, maintenance of myocardial hypothermia to reduce the metabolic demand, avoidance of prolonged aortic cross-clamp times and prevention of extended LV distention - which may be particularly challenging to detect in the setting of minimally-invasive procedures - to preserve myocardial viability [3, 4]. Another (uncommon) protective measure suggested in literature is the intravenous administration of propranolol prior to aortic cross-clamp [3, 4, 7]. Moreover, an in vitro study published in 1980 further suggested intermittent brief ventricular stretching during cardioplegic arrest to prevent contracture, although this concept has never been evaluated in vivo and somehow conflicts with the idea of meticulous LV decompression to prevent LV distention [8]. 

Once diagnosed, treatment options are extremely limited [2]. Curative treatment may only be achieved by the use of short- or long-term mechanical circulatory support as bridging strategy and listing for high-urgency cardiac transplantation in carefully selected patients, with the timing of escalation largely depending on the individual clinical course and characteristics as well as evidence of meaningful myocardial recovery.

## Data Availability

No datasets were generated or analysed during the current study.

## Abbreviations

ECMELLA: Extracorporeal membrane oxygenation and Impella
ECMO: Extracorporeal membrane oxygenation
HTK: Histidine-tryptophan-ketoglutarate
LV: Left ventricular
LVAD: Left ventricular assist device
MV: Mitral valve
POD: Postoperative day
Va: Veno-arterial
Vav: Veno-arterio-venous
